# Introducing the Newly Isolated Bacterium *Aneurinibacillus* sp. H1 as an Auspicious Thermophilic Producer of Various Polyhydroxyalkanoates (PHA) Copolymers–2. Material Study on the Produced Copolymers

**DOI:** 10.3390/polym12061298

**Published:** 2020-06-05

**Authors:** Petr Sedlacek, Iva Pernicova, Ivana Novackova, Xenie Kourilova, Michal Kalina, Adriana Kovalcik, Martin Koller, Jana Nebesarova, Vladislav Krzyzanek, Kamila Hrubanova, Jiri Masilko, Eva Slaninova, Monika Trudicova, Stanislav Obruca

**Affiliations:** 1 Faculty of Chemistry, Brno University of Technology, Purkynova 118, 612 00 Brno, Czech Republic; sedlacek-p@fch.vut.cz (P.S.); xcpernicovai@fch.vut.cz (I.P.); xcnovackova@fch.vut.cz (I.N.); xckourilovax@fch.vut.cz (X.K.); kalina-m@fch.vut.cz (M.K.); kovalcik@fch.vut.cz (A.K.); masilko@fch.vut.cz (J.M.); xcslaninovae@fch.vut.cz (E.S.); xctrudicova@fch.vut.cz (M.T.); 2 Institute of Chemistry, NAWI Graz, University of Graz, Heinrichstrasse 28/VI, 8010 Graz, Austria; martin.koller@uni-graz.at; 3 ARENA Arbeitsgemeinschaft für Ressourcenschonende & Nachhaltige Technologien, Inffeldgasse 21b, 8010 Graz, Austria; 4 Biology Centre, The Czech Academy of Sciences, v.v.i., Branisovska 31, 370 05 Ceske Budejovice, Czech Republic; nebe@paru.cas.cz; 5 Faculty of Science, University of South Bohemia, Branisovska 31, 370 05 Ceske Budejovice, Czech Republic; 6 Institute of Scientific Instruments of the Czech Academy of Sciences, v.v.i., Kralovopolska 147, 612 64 Brno, Czech Republic; krzyzanek@isibrno.cz (V.K.); hrubanova@isibrno.cz (K.H.)

**Keywords:** polyhydroxyalkanoates, thermophiles, *Aneurinibacillus* sp., P(3HB-*co*-4HB), P(3HB-*co*-3HV-*co*-4HB), crystallinity

## Abstract

*Aneurinibacillus* sp. H1 is a promising, moderately thermophilic, novel Gram-positive bacterium capable of the biosynthesis of polyhydroxyalkanoates (PHA) with tunable monomer composition. In particular, the strain is able to synthesize copolymers of 3-hydroxybutyrate (3HB), 4-hydroxybutyrate (4HB) and 3-hydroxyvalerate (3HV) with remarkably high 4HB and 3HV fractions. In this study we performed an in-depth material analysis of PHA polymers produced by *Aneurinibacillus* sp. H1 in order to describe how the monomer composition affects fundamental structural and physicochemical parameters of the materials in the form of solvent-casted films. Results of infrared spectroscopy, X-ray diffractometry and thermal analysis clearly show that controlling the monomer composition enables optimization of PHA crystallinity both qualitatively (the type of the crystalline lattice) and quantitatively (the overall degree of crystallinity). Furthermore, resistance of the films against thermal and/or enzymatic degradation can also be manipulated by the monomer composition. Results of this study hence confirm *Aneurinibacillus* sp. H1 as an auspicious candidate for thermophilic production of PHA polymers with material properties that can be tuned together with their chemical composition by the corresponding adjustment of the cultivation process.

## 1. Introduction

Polyhydroxyalkanoates (PHA) represent the family of polyesters of hydroxyalkanoic acids under consideration, being renewable and biodegradable alternatives to petrochemical polymers [1]. These materials are accumulated by various bacteria and Archaea in the form of intracellular granules primarily serving as storage materials. Nevertheless, it was recently reported that these materials also enhance stress robustness and the resistance of microbes against a wide range of stress factors [2].

It is noteworthy that PHA in native intracellular granules are completely amorphous revealing the properties of super-cooled liquids. This unique, thermodynamically unfavorable state of the polymers is enabled by the synergy effects of (i) the presence of intergranular water which acts as a plasticizer and (ii) the small granular diameters, since crystallization is substantially inhibited in small volumes of native PHA granules [3]. It seems that the unique biophysical properties of amorphous PHA granules protect bacteria from a wide range of environmental stressors such as freezing-thawing events [4], osmotic pressure [5] or UV radiation [6]. However, when the native structure of the PHA granules is damaged, e.g., if the PHA are extracted from bacterial cells, the polymers quickly crystalize [3], which is accompanied by substantial changes in their material properties.

Material characteristics of PHA are strongly dependent upon monomer composition. PHA can be classified as short-chain-length (scl-PHA) if their monomer units consist of 3–5 carbon atoms, or medium-chain-length (mcl-PHA), which indicates that monomer units contain 6–14 carbons. Generally, scl-PHA are stiff thermoplastic polymers with a high degree of crystallinity and typically pronounced brittleness, while mcl-PHA reveals a much lower degree of crystallinity and ductility [7]. Nevertheless, even among scl-PHA there are great differences in properties. Poly(3-hydroxybutyrate) (P(3HB)), the homopolymer of 3-hydroxybutyrate (3HB), is the most common member of the PHA family; P(3HB) is highly crystalline and brittle with a melting temperature of about 180 °C, which is very close to its temperature of degradation (approx. 200 °C). However, when other monomer units such as 3-hydroxyvalerate (3HV) or 4-hydroxybutyrate (4HB) are introduced into the polymer chain, the melting temperature as well as the crystallinity of the material are substantially reduced, which is a very positive feature for the processing of the material and ultimately for numerous applications of these polymers [8]. In particular, copolymers containing high fractions of 4HB are considered as auspicious materials for various high-value applications, including but not limited to medicine and the health-care sector [9].

We have recently isolated the novel thermophilic bacterium *Aneurinibacillus* sp. H1 [10], which demonstrated extraordinary potential for PHA production. In general, biotechnological processes based on extremophilic bacteria are considered very promising since these bacteria are naturally resistant to microbial contamination which reduces operation costs and increases their efficiency as whole-cell biocatalysts [11]. *Aneurinibacillus* sp. H1 is a Gram-positive bacterium with optimal growth temperature between 45–50°C, and capable of producing various scl-PHA polymers. When cultivated on glucose or glycerol, it accumulates the homopolymer P(3HB). However, when supplemented by suitable precursors such as 1,4-butanediol (1,4-BD) or valerate, it is capable of introducing 4HB or 3HV subunits into the polymer chain, respectively, resulting in production of copolymers poly(3-hydroxybutyrate-*co*-4-hydroxybutyrate) (P(3HB-*co*-4HB)) and poly(3-hydroxybutyrate-*co*-3-hydroxyvalerate) (P(3HB-*co*-3HV)) or even the terpolymer poly(3-hydroxybutyrate-*co*-3-hydroxyvalerate-*co*-4-hydroxybutyrate) (P(3HB-*co*-3HV-*co*-4HB)). The bacterium is able to synthesize PHA with very high portions of 4HB and 3HV units, and the monomer composition of PHA can be simply controlled by manipulating the composition of cultivation media.

Our previous work was dedicated to the evaluation of the biotechnological potential of *Aneurinibacillus* sp. H1 with respect to production of various PHA copolymers (submitted for publication). In this work we focused on the in-depth material characterization of PHA polymers produced by *Aneurinibacillus* sp. H1. To provide a general view on how the incorporation of 4HB monomer units changed the material properties of the polymer, we also included the homopolymer P(3HB) and P(3HB-*co*-4HB) copolymers with a low content of 4HB, produced by the routinely used PHA producing bacterium *Cupriavidus necator* H16, in the study.

## 2. Materials and Methods

### 2.1. Production of PHA in Shaking Flasks by Aneurinibacillus sp. H1

The inoculum was grown in complex media nutrient broth (10 g/L beef extract, 10 g/L peptone, 5 g/L NaCl) at 45 °C with shaking at 190 rpm. For production of PHA, mineral salt medium (MSM) was used consisting of: Na_2_HPO_4_·12H_2_O, 9.0 g/L; KH_2_PO_4_, 1.5 g/L; MgSO_4_·7H_2_O, 0.2 g/L; NH_4_NO_3_, 1.0 g/L; CaCl_2_·2H_2_O, 0.02 g/L; Fe^III^NH_4_citrate, 0.0012 g/L; Tryptone, 0.5 g/L with 1 mL/L of MES. Glycerol was used as the main carbon substrate, 1,4-BD was applied as precursor of 4HB. To gain various fractions of 4HB in polymers, glycerol and 1,4-BD were applied at various concentrations and ratios: glycerol 8 g/L + 4 g/L 1,4-BD resulted in a copolymer with 36 mol.% of 4HB, glycerol 6 g/L + 4 g/L 1,4-BD provided a copolymer with 42 mol.% of 4HB, glycerol 4 g/L + 4 g/L 1,4-BD gained a polymer with 74% of 4HB, glycerol 2 g/L + 4 g/L 1,4-BD yielded a polymer with 84% of 4HB, and by application of 4 g/L of 1,4-BD, the 4HB fraction achieved 90 mol.%. Valerate (2 g/L) was used as a precursor of 3HV for terpolymer production. The inoculum ratio was 10 vol.%. Production cultivations were carried out for 72 h at 45°C under constant shaking of 180 rpm. All the cultivations were performed in duplicate. After cultivation, bacterial cells were harvested by centrifugation (6000× *g*, 5 min). Biomass was determined gravimetrically as the cell dry mass (CDM), and the amount and monomer composition of PHA were analyzed by Gas Chromatography as reported previously [12].

### 2.2. Production of P(3HB-co-4HB) Copolymers with Low 4HB Fraction Employing Cupriavidus necator H16

To gain P(3HB-*co*-4HB) copolymers with low fraction of 4HB we employed the well-described mesophilic PHA producer *Cupriavidus necator* H16 (CCM 3726). The production was carried out in Erlenmeyer flasks (volume 250 mL) containing 100 mL of mineral medium (composition: (NH_4_)_2_SO_4_, 3 g/L; KH_2_PO_4_, 1 g/L; Na_2_HPO_4_·12H_2_O, 11.1 g/L; MgSO_4_, 0.2 g/L; 1 mL of microelement solution and 1 L of distilled water. The microelement solution was composed of FeCl_3_, 9.7 g/L; CaCl_2_·2H_2_O, 7.8 g/L; CuSO_4_·5H_2_O, 0.156 g/L; CoCl_2_·6H_2_O, 0.119 g/L; NiCl_2_, 0.118 g in 1 L of 0.1 M HCl. The cultivation setup was inoculated with 5 mL of overnight cultures grown in nutrient broth medium. To induce production of P(3HB) homopolymer, we used fructose (20 g/L) as a sole carbon source, production of P(3HB-*co*-4HB) copolymer was induced by application of fructose (8 g/L) and γ-butyrolactone (GBL) (2 and 6 g/L). After 72 h of cultivation, the cells were harvested (centrifugation, 8000× *g*, 5 min) and the biomass, PHA content and composition of PHA was determined as described above.

### 2.3. Preparation of PHA Films by Solvent Casting

PHA was extracted from approximately 100 mg of dried biomass by 5 mL of chloroform at 70°C for 24 h. Afterwards, residual bacterial biomass was removed by filtration, and the chloroform phase containing dissolved PHA was poured onto glass Petri dishes (3 cm in diameter), and the solvent was allowed to evaporate at laboratory temperature overnight. Obtained films were stored in the dark to avoid light-induced polymer degradation, and subjected towards determination of material properties.

### 2.4. Characterization of PHA Films

#### 2.4.1. Molecular Weight Determination by Size Exclusion Chromatography (SEC-MALS)

The molecular weight of the polymers was accessed as follows: 5 mg of the polymer was solubilized in 1 mL of HPLC-grade chloroform and the obtained samples were passed through syringe filters (nylon membrane, pore size 0.45 mm) and analyzed by gel size exclusion chromatography (Agilent, Santa Clara, CA, USA, Infinity 1260 system containing PLgel MIXED-C column) coupled with multiangle light scattering (Wyatt Technology, Dawn Heleos II, Santa Barbara, CA, USA) and differential refractive index (Wyatt Technology, Optilab T-rEX, Santa Barbara, CA, USA) detection. For the analysis, 100 mL of individual samples were injected into the chromatographic system containing HPLC-grade chloroform (pre-filtered through 0.02 mm membrane filter) as mobile phase. The used flow rate was 0.6 mL/min. The weight-average molecular weight (Mw) and polydispersity index (PDI, ratio of weight-average and number-average molecular weight Mw/Mn) were determined using ASTRA software (Wyatt Technology, version 6.1, Santa Barbara, CA, USA) based on Zimm’s equations. The used value of refractive index increment (dn/dc) for P(3HB) was 0.0336 mL/g. This value was determined from the differential refractometer response assuming a 100% sample mass recovery from the column.

#### 2.4.2. Attenuated Total Reflectance Fourier-Transform Infrared (ATR-FTIR) Spectrometry

ATR FTIR spectra of the PHA films were collected with iS50 FTIR spectrometer (Thermo Scientific, Waltham, MA, USA). All measurements were taken from a sample surface at ambient temperature (in an air-conditioned room) on the built-in single-reflection diamond attenuated total reflectance (ATR) crystal. An individual absorption spectrum was collected as an average of 16 scans with a resolution of 1/cm. Every sample was measured at 10 different spots on its surface.

#### 2.4.3. X-ray Diffractometry (XRD)

XRD diffraction patterns were collected using the X-ray diffraction analyzer EMPYREAN (PANalytical, Malvern, United Kingdom) in a central focusing arrangement with Bragg-Brentano parafocusing optics using CuKα radiation (range: 5–90°2th, step: 0.013°2th, voltage: 40 kV, current 30 mA), ADS: 10 mm, time per step: 96 s, without monochromator.

#### 2.4.4. Methods of Thermal Analysis

Differential scanning calorimetry (DSC) was performed using a temperature-modulated calorimeter (DSC Q2000, TA Instruments, New Castle, DE, USA) equipped with an RCS90 cooling accessory. All experiments were performed in hermetically sealed TzeroTM (TA Instruments, Lukens, DE, USA) aluminum pans under a dynamic nitrogen atmosphere. Temperature-modulated DSC was applied in order to investigate the melting-crystallization behavior of the PHA films. Samples of a mass of approximately 5 µg were first equilibrated at 200 °C and then cooled down at a cooling rate of 2 °C/min and a temperature modulation of ±0.6 °C every 60 s. After another equilibration step (10 min at −80 °C), the sample was heated to 200 °C with an underlying heating rate of 2 °C/min and the same temperature modulation. Evaluation of the thermograms was performed by the TAUniversal Analysis 2000 software (TA Instruments, Lukens, DE, USA). In order to distinguish between individual reversible and irreversible processes (e.g., melting vs. cold crystallization), the total heat flow was divided into reversible and irreversible components, and the two respective thermograms were evaluated separately.

The thermogravimetry analyzer TGA Q5000 (TA Instruments, New Castle, DE, USA) was used to determine the mass loss in the temperature interval 25–600 °C under a dynamic dry air atmosphere with a heating rate of 10 °C/min. 

#### 2.4.5. Enzymatic Degradation Assay

The biodegradability of the tested materials was determined using two different enzymes: extracellular PHA depolymerase capable of hydrolysis of crystalline PHA. This enzyme was obtained as a supernatant after cultivation of *Schlegelella thermodepolymerans* DSM 15344 in medium consisting of 9 g/L Na_2_HPO_4_·12H_2_O; 1.5 g/L KH_2_PO_4_; 1 g/L NH_4_Cl; 0.2 g/L MgSO_4_·7H_2_O; 0.02 g/L CaCl_2_·2H_2_O; 0.0012 g/L NH_4_Fe^III^citrate; 0.5 g/L yeast extract; 1 mL/L trace elements solution (TES). Composition of TES: 50 g/L EDTA; 8.3 g/L FeCl_3_; 0.84 g/L ZnCl_2_; 0.13 g/L CuCl_2_·2H_2_O; 0.1 g/L CoCl_2_·6H_2_O; 0.016 g/L MnCl_2_·6H_2_O; 0.1 g/L H_3_BO_3_. Cultivations were performed for 72 h at 50 °C (180 rpm) using glycerol as the sole carbon source. In addition, also an intracellular depolymerase of *Bacillus subtilis* WB800 specific for amorphous PHA was expressed and purified as described by Hermawan and Jendrossek [13].

The biodegradation assay was performed using thin films of approximately 5 mg poured in 5 mL of 50 mM phosphate buffer (pH 7.4) with above described PHA depolymerases. Incubation was performed for 3 h at 37 °C for *Bacillus subtilis* PHA depolymerase, or for 24 h at 50 °C for *Schlegelella thermodepolymerans* depolymerase.

Micrographs of the original and partially degraded films were recorded using Zeiss EVO LS-10 scanning electron microscope (SEM) (Carl Zeiss Ltd., Cambridge, UK). Before the analysis, a film cut-off was stuck on a carbon tape and sputter-coated with gold.

## 3. Results and Discussion

### 3.1. Monomer Composition and Molecular Weight

The isolated thermophilic strain *Aneurinibacillus* sp. H1 is capable of production of PHA copolymers with a great variety of accessible monomer compositions resulting from the proper adjustment of cultivation conditions. As no special cultivation protocol for block copolymer biosynthesis described in [8] was adopted in this study, we expected that in all the produced copolymers, monomer units would be randomly distributed in heteropolymer chains. In order to evaluate the relationship between the monomer composition and fundamental application-relevant properties of the resulting PHA materials, selected PHA copolymers produced by the strain were solvent-casted onto compact solid films and subjected to complex structural and physicochemical characterization. Furthermore, in order to cover the whole range of the accessible relative content of 3HB and 4HB monomers in P(3HB-*co*-4HB), in this material analysis we also included three reference materials with no or a very low content of 4HB in the polymer chains, produced by the routinely used mesophilic PHA producer *C. necator* H16. A complete list of materials included in this assay, together with their monomer compositions and molecular weights, is presented in Table 1, a visual illustration of the appearance of the films is provided in Supplementary Materials Figure S1.

The molecular weight values of the PHA samples with low 4HB portions produced by *C. necator* were substantially higher than that of polymers produced by *Aneuribacillus* sp. H1 containing high 4HB fractions. This difference could be, of course, caused by biological dissimilarities between the producing strains; nevertheless, it was a common feature that incorporation of a high fraction of 4HB was associated with a decrease in Mw of PHA, such an effect was observed in *Cupriavidus* sp. USMAA1020 [14] and *Alcaligenes* sp. A-04 [15]. We can hypothesize that 4HB-CoA is a less suitable substrate for PHA synthase than 3HB-CoA and, therefore, its more frequent incorporation into PHA chains is associated with a presumable termination of polymerization needed for PHA chain growth, resulting in lower Mw of polymer. However, it could also be seen that in material produced by *Aneurinibacillus* the Mw of the polymer increased with the increase in 4HB content. The explanation could be that glycerol was used as the second substrate along with 1,4-BD. The higher the portion of glycerol applied, the lower the portion of 4HB obtained, with a decreased Mw. Generally, PHA produced on glycerol revealed lower Mw since glycerol terminated the synthesis of PHA chains by the “endcapping effect” [16], and it was likely that the polymerization-terminating effect of glycerol was more pronounced than that of 4HB-CoA. In addition, there is another possible explanation why the Mw of polymer produced by *Aneurinibacillus* sp. H1 is substantially lower than that produced by *C. necator*: *C. necator* possesses a PHA synthase consisting of only one PhaC subunit. In contrast, PHA synthases of bacilli and related species, including members of the genus *Aneurinibacillus,* consist of two different subunits, PhaC and PhaR. It was recently reported that PhaR subunits catalyzed alcoholysis of the PHA chain when some alcohols were present in cultivation media [17]. Therefore, both substrates used for cultivation of *Aneurinibacillus* sp. H1–glycerol and 1,4-butanediol might be involved in this reaction, catalyzed by PhaR, which could substantially contribute to the fact that PHA produced by *Aneurinibacillus* sp. H1 reveals substantially lower Mw as compared to the polymer synthesized by *C. necator*.

### 3.2. Chemical and Physical Structure Determined by FTIR Spectra 

**Figure 1:**
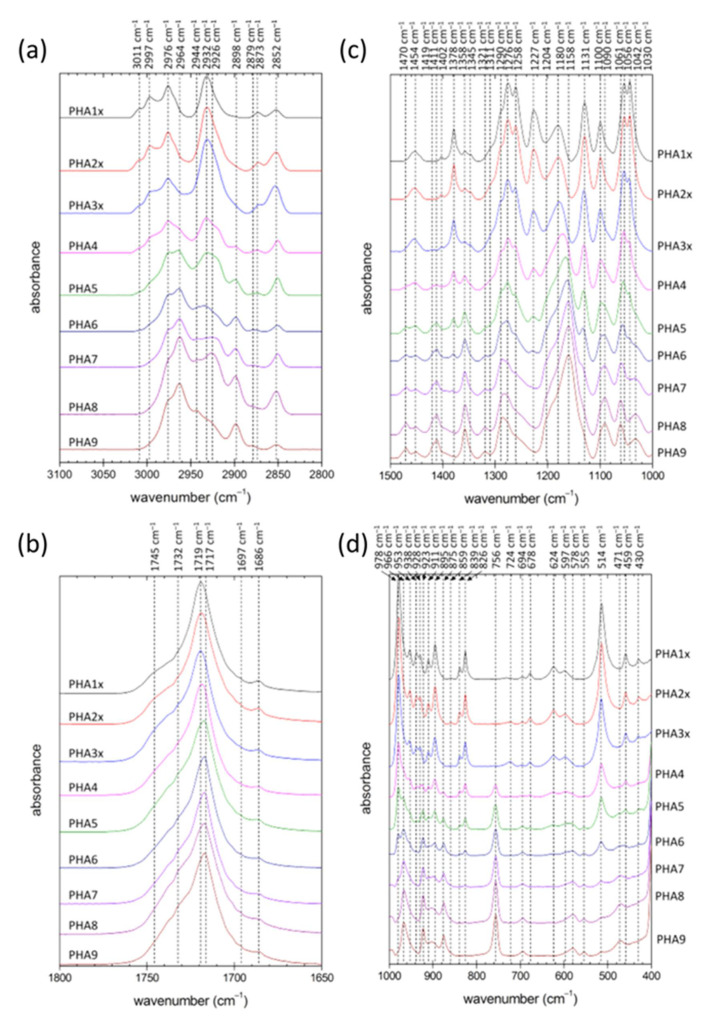
Normalized ATR FTIR spectra (cut-offs for the analytically important spectral regions) of the solution-casted PHA films prepared from polymers with different monomer composition (each spectrum represents an arithmetic average of 10 individual spectra collected at different locations on the surface of the film). Frequencies of the absorption band maxima were determined from the second derivative of the spectra.

The study of the variation in chemical structure of the PHA copolymer films was followed by ATR FTIR spectrometry. To deal with potential surface heterogeneity of the films, for an individual film 10 separate spectra were taken at different spots on the film surface and averaged. Figure 1 shows comparison of the normalized spectral cut-offs of the average spectra in analytically important spectral regions for all tested materials (complete spectra are provided as Supplementary Materials Figure S2).

As expected, FTIR spectra clearly illustrated differences in the chemical composition of the individual polymer materials. This was obvious mainly from the changes in characteristic absorption of alkyl groups (note, e.g., the varying intensity of methyl absorption at 1258 and 1378 cm^−1^, and differences in relative intensity of methyl and methylene absorptions at around 2873 and 2852 cm^−1^, respectively). Nevertheless, further analysis of the FTIR spectra could also provide additional information regarding the degree of structural order in the tested PHA materials. Utilization of FTIR spectroscopy for evaluating PHA crystallinity has been proposed by several authors [3,18,19,20]. 

All PHA materials tested in this study showed spectral signs of semi-crystalline solids combining the spectral attributes of crystalline and amorphous structural motifs. This was obvious for instance from the typical asymmetrical shape of the carbonyl C=O vibration band (see Figure 1b), which in fact represented a spectral envelope of the overlapping vibration bands of PHA ester carbonyls in ordered (<1725 cm^−1^) and disordered (about 1740 cm^−1^) structures. Commonly, the FTIR-based crystallinity assay focuses on analysis of vibration modes specifically assigned to structural groups located either in crystalline or in amorphous polymer domains. In the case of PHA, absorption bands of ester groups are most often analyzed for this purpose. In the homopolymer P(3HB), the vibration band at 1180 cm^−1^ was attributed to amorphous PHA domains, while the characteristic conformational bands of the crystalline phase were those at 1276 and 1227 cm^−1^, where the latter was specifically assigned to helical crystallites. It can be seen in Figure 1c that the intensity of both bands decreased with the decreasing relative content of 3HB in the polymer structure (note the significant shift in intensities of these two bands compared to the 1180 cm^−1^ band for the low 4HB content of 6.5% in PHA3x). Nevertheless, while the band at 1276 cm^−1^ was still present in the spectra of materials rich in 4HB, the 1227 cm^−1^ band was missing for a content of 3HB lower than about 20 wt.% (samples PHA7, PHA8 and PHA9). This confirmed a well-known fact that in 3HB-poor PHA chains, ordered structural motifs could still be found, but their structure was different than that of P(3HB) crystallites [21]. Disappearance of ordered structures specific for P(3HB) could also be tracked by changes in the C–H stretching region (lower relative intensity of blue-shifted antisymmetric stretching bands at about 3000 cm^−1^) and in the C–C stretching region (e.g., disappearance of bands assigned to P(3HB) crystallites at 978 cm^−1^ and specific vibration mode of the helical structure at 826 cm^−1^). 

On the other hand, an increasing content of the monomer 4HB in the polymer chains gave rise to another ordered structure with its own signature in the FTIR spectra. The asymmetric C–H stretching band of methylene groups was blue-shifted from 2932 to 2964 cm^−1^ in crystalline structures (see Figure 1a). Similarly, the vibration band at 2898 cm^−1^ could be assigned to blue-shifted symmetric stretching of methylene groups in ordered domains. Similar spectral signs of crystallization were described for other PHA polymers such as poly(3-hydroxybutyrate-*co*-3-hydroxyhexanoate) [20] and their structural analogues of, e.g., poly(ε-caprolactone) (PCL) [22] or polyglycolic acid (PGA) [23]. Furthermore, methylene deformation bands were known to be sensitive to chain conformation as well. The main CH_2_ deformation modes including methylene scissoring (1470 cm^−1^), methylene wagging (1321 cm^−1^) and the bending deformation that formed an absorption shoulder at 1204 cm^−1^ increased in intensity when associated with the trans isomer, which was more abundant in stretched polymer chains found in the crystalline structures. Similarly, the P(3HB) crystallites, and the ordered structure built up on the 4HB monomer, the vibration modes attributed to the ester bond might be taken as a crystallinity marker. Phillipson et al. noted that for PCL, stretching C–O bands at 1235 and 1275 cm^−1^ were blue-shifted on crystallization to 1245 and 1295 cm^−1^, respectively. Apparently, a similar phenomenon could be observed for PHA copolymers rich in 4HB monomers, where shoulders at about 1250 and 1290 cm^−1^ were increasing in intensity with the relative content of 4HB. Similarly, the bending mode of C–O–C (966 cm^−1^), which was intensive in copolymers rich in 4HB, was known to strengthen on crystallization of PCL [22]. 

To sum up the above discussed results of ATR FTIR analysis, it was shown that gradual substitution of 3HB monomer units by 4HB resulted firstly in a decrease in the degree of structural order in the material as the relative content of P(3HB) crystallites decreased, but, for higher content of 4HB, the crystallinity increased again as the P(4HB) crystallites started to predominate the structure; a switch from a P(3HB) to a P(4HB) lattice was observed. P(3HB) and P(4HB) crystallites are hence not structurally isomorphic, i.e., a crystallite cannot incorporate the other monomer into its structure without its significant deformation. This incompatibility of 3HB and 4HB monomers has already been described [21] and represents a promising tool for manipulating the overall crystallinity of PHA materials via controlling their monomer composition. Contrarily, the 3HV monomer could be incorporated into the P(3HB) lattice to some extent, thus showing some isomorphism of the P(3HB) and P(3HV) lattice [21]. Nevertheless, the terpolymer PHA9 (54% 4HB, 33% 3HV, 13% 3HB) manifested all the spectral features discussed above for 4HB-dominating copolymers. Apparently, the 3HB and 3HV content was so low that no crystallites of P(3HB) or the structurally similar P(3HV) lattice were formed together with the P(4HB) crystallites.

ATR FTIR analysis provided valuable but limited information on the crystallinity of the tested materials. The technique provided neither any detailed information on the morphology of the crystallites nor a direct quantification of the overall crystallinity of the material. For these purposes, the films were further subjected to X-ray diffractometry (XRD) and differential scanning calorimetry (DSC).

### 3.3. Detailed Crystallinity Assay Provided by XRD and DSC

**Figure 2:**
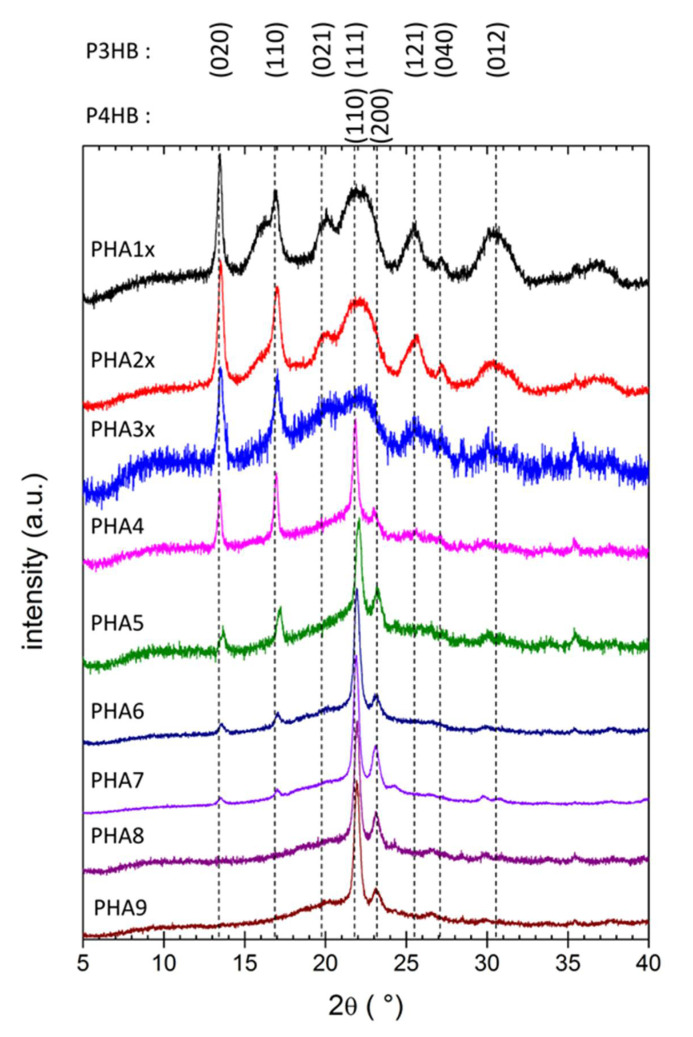
X-ray diffraction patterns of copolymer PHA films. Diffractograms were normalized for clarity. Reflections are assigned to P(3HB) and P(4HB) lattices, respectively, according to Gao et al. [25] and Keridou et al. [27].

The XRD patterns of all the tested films are shown in Figure 2. Again, all materials showed the characteristic features of semicrystalline solids as the XRD patterns combined the specific diffractions of ordered structural motifs with a broad background amorphous halo. The shape of the XRD patterns and position of diffraction maxima were in expedient correspondence with published XRD features of the P(3HB) and the P(4HB) lattice [21,24,25,26,27]. Individual diffraction maxima were assigned to the corresponding reflections of the two crystal lattices in Figure 2. 

The XRD patterns in Figure 2 were normalized for clarity of their comparison, and the overall crystallinity or amorphous content could therefore hardly be quantified from the figure. Nevertheless, from the qualitative viewpoint, it could clearly be seen that the relative content of P(3HB) and P(4HB) crystallites was gradually shifted with the corresponding change in monomer composition. The P(3HB) crystal lattice was the dominating structure for polymers with a low content of 4HB monomers (PHA1x, PHA2x, PHA3x). In this crystal lattice, the helical form of the polymer chain dominated (represented by sharp diffractions (020) and (110)), but stretched β-structures are present as well (diffraction (021)). For the copolymers with intermediate content of 4HB monomer (36% and 42% in this study), strong diffractions of both (P(3HB) and P(4HB)) crystal lattices could be found in the XRD pattern. This was in good agreement with results of FTIR analysis as far as the spectral signatures of both types of ordered structures were detected in ATR FTIR spectra of these copolymers. On the other hand, diffractions of the P(3HB) lattice were detected in the XRD pattern up to 4HB content as high as 84%. On the one side, this confirms that the XRD technique is more sensitive than FTIR for the qualitative description of PHA’s crystallinity (no apparent spectral signs of P(3HB)’s crystalline structures were found in the FTIR spectrum of the copolymer PHA7 with 84% 4HB). On the other hand, it contradicts the observations of Saito [24], who had not detected any diffractions of the P(3HB) lattice in XRD patterns of copolymers with a 4HB content higher than 64%. Similar to FTIR, the terpolymer PHA9 provided XRD features similar to P(3HB-*co*-4HB) copolymers with 4HB being the dominating component.

In general, the results of XRD analysis confirmed the qualitative conclusions of the FTIR-based crystallinity assay. It could be seen that, at a low content, the 4HB monomer acted as a structure-breaking component. This was in agreement with previous works reporting that even if both (P(3HB) and P(4HB)) lattices have orthorhombic unit cells with the space group P2_1_2_1_2_1_ [21,26,27], differences in the spacings of the two-unit cells were so large that the 4HB unit could not crystallize in the sequence of 3HB monomers and formed a defect in the P(3HB) crystal lattice [21]. In the same perspective, when the 4HB monomer predominated the polymer composition, residual 3HB units reduced the overall P(4HB) crystal lattice content in a similar way. Different structural effects were described in literature for the presence of 3HV units in PHA copolymers [21]. In the P(3HB-*co*-3HV) copolymers, P(3HB) to P(3HV) crystal lattice transition occurred at 3HV content of approximately 40%. Furthermore, contrarily to 4HB, 3HV units could be incorporated into the P(3HB) lattice (and vice versa), and the negative effect of the monomer on the overall crystallinity was therefore smaller. Nevertheless, for the terpolymer tested in this study (PHA9), the content of 3HB and 3HV were too low to let the P(3HB) or P(3HV) crystallites form.

**Figure 3:**
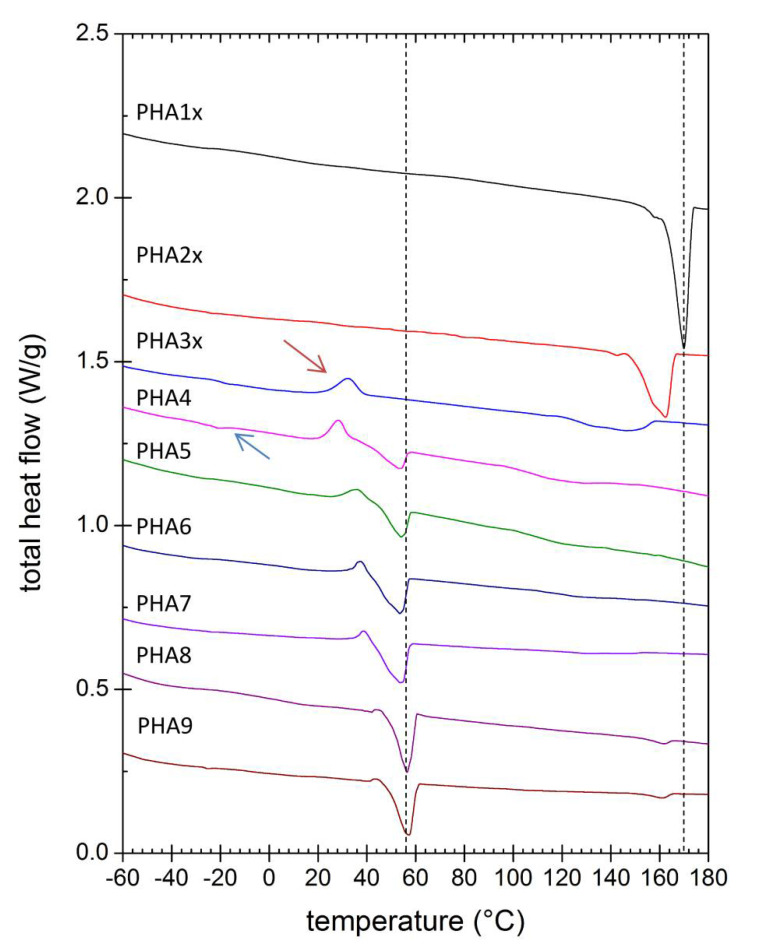
Differential scanning calorimetry (DSC) thermograms of PHA copolymer films (thermograms show the second heating scan).

The melting behavior of the solvent-casted PHA films was studied using DSC. In order to evaluate the intrinsic tendency of the polymer to crystallize (unaffected by the potential artifacts of the film preparation procedure), all samples were first of all melted and cooled down with the same heating/cooling program to erase the polymers’ thermal history. The second heating scan was then analyzed (corresponding thermograms are shown in Figure 3). It could be seen that while the homopolymer P(3HB) melted at about 170 °C, even a very low content of 4HB reduced the melting point dramatically. Furthermore, the initially sharp melting endotherm of P(3HB) broadened when a low content of 4HB was present. This further illustrated the above mentioned structure-breaking effect of 4HB monomers resulting in P(3HB) crystalline domains being reduced both in number and in size (decreasing size of P(3HB) crystallites could also be deduced from the widening diffraction peaks in XRD patterns; note, e.g., the different widths of diffraction peaks for PHA1x and PHA3x). Polymers of intermediate 4HB content (PHA4, PHA5) showed two melting endotherms, indicating again the presence of both types of crystallites (P(3HB), P(4HB)). For these samples, a higher content of the amorphous phase was manifested by the most pronounced glass transition at temperature about −20 °C (T_g_; marked with blue arrow in Figure 3). The other prepared PHA samples also possessed a T_g_, but DSC was not a sufficiently sensitive method to determine the T_g_ for semi-crystalline polymers with a high degree of crystallinity. Therefore, the appearance of T_g_ on the DSC thermogram might indicate a higher proportion of the amorphous phase in PHA compared to other samples. Copolymers with low and intermediate content of 4HB also showed an exothermic peak during the heating step, which represented the cold crystallization of the polymer chains (red arrow in Figure 3). P(3HB-*co*-4HB) copolymers with the highest content of 4HB melted at about 55 °C, where the increasing residual content of 3HB decreased the melting point again. Similar to the results of the other material analysis methods, the presence of 3HV in the terpolymer PHA9 virtually amplified the effects of 4HB (the material behaved like it had a higher 4HB content).

**Figure 4:**
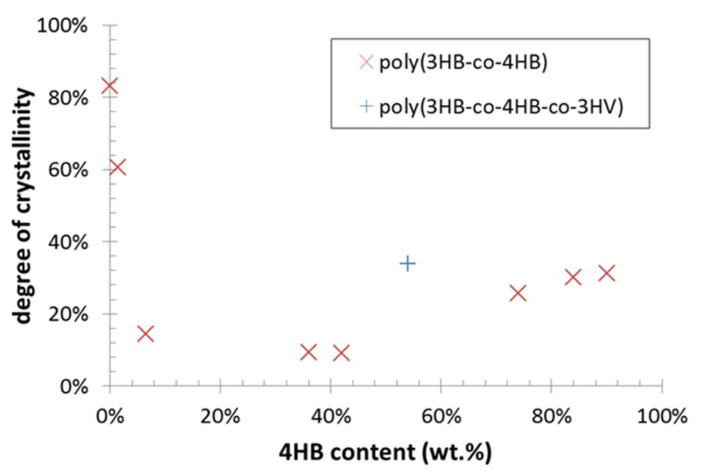
Degree of crystallinity of PHA films estimated from DSC analysis.

Total intrinsic crystallinity of the polymers was estimated from the second heating scan. For this purpose, specific heat of fusion was calculated separately for P(3HB) and P(4HB) crystallinity by integrating corresponding melting endotherms. Furthermore, the undesirable crystallinity overestimation caused by the cold crystallization was eliminated by integration of the cold crystallization exotherm and subtracting the corresponding integral from the total melting enthalpy. Values of the standard heat of fusion for 100% crystalline P(3HB) (146 J/g) and for 100% crystalline P(4HB) (110 J/g) were taken from literature [24,28]. For the terpolymer (sample PHA9), heat of fusion for P(3HB) lattice was applied on the high-temperature endotherm even if it was not possible to attribute the lattice structure of this type of crystallites unambiguously. Nevertheless, this simplification could be justified, taking into account the high structural similarity of P(3HB) and P(3HV) crystallites and low differences between heat of fusion values of P(3HB) and P(3HV) lattices [29], a melting point too close to that of PHA homopolymer (<10 °C melting point shift) than expected for P(3HV) lattice [21] and also the minor relative contribution of the high-temperature endotherm to the total specific heat (approximately 11% of the total specific heat). Dependence of the total crystallinity on the 4HB content in the polymer composition is shown in Figure 4. Note that the results in the figure might still be affected by some additional systematic errors (e.g., temperature dependency of the heat of fusion or dynamic effects linked to the non-zero heating rate were not taken into account). Nevertheless, they provide an intriguing illustration of the U-shaped crystallinity-composition function of PHA copolymers, which seems to be a general material feature of PHA copolymers [21].

### 3.4. Stability Against Thermal and Enzymatic Degradation

The above discussed results of material analyses confirm that controlling the monomer composition of PHA polymers enables the tailoring of the type and degree of ordering in the polymer structure. This represents a crucial material quality that influences the mechanical, thermal and chemical performance of the final products. To verify this connection, we performed preliminary stability tests dealing with resistance of the solvent-casted PHA films against thermal and enzymatic degradation.

**Figure 5:**
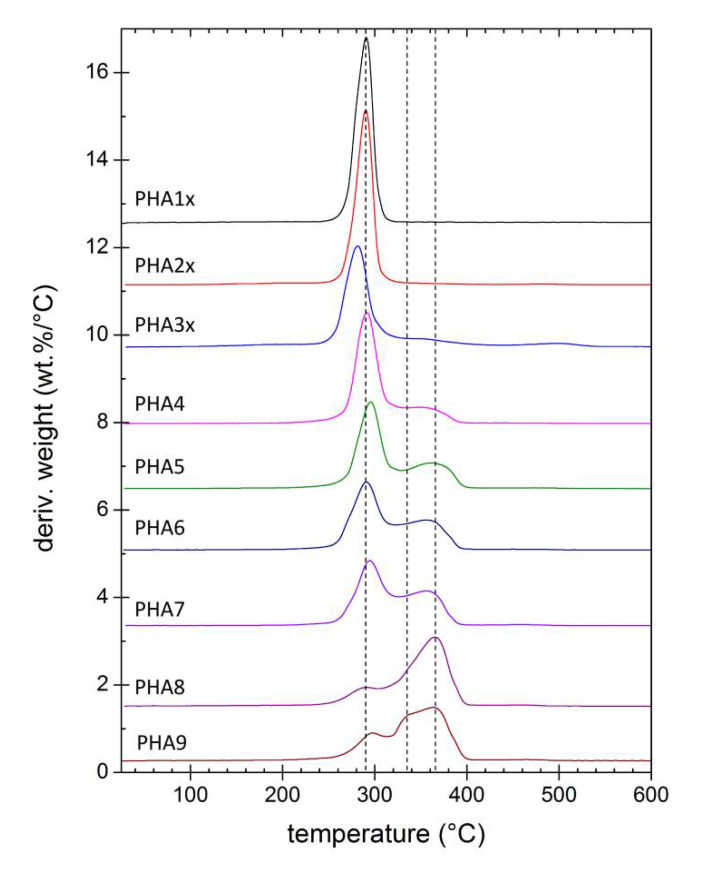
DTG curves of solvent-casted films from the tested PHA copolymers.

**Figure 6:**
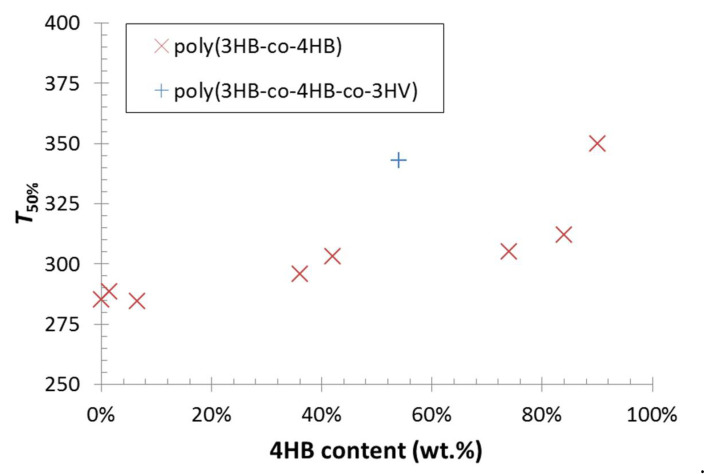
Half-decomposition temperatures (T_50%_; temperature corresponding to 50% residual weight) as a function of 4HB content in the polymer structure.

Thermal degradation of tested PHA materials was followed by thermogravimetry. Figure 5 provides derivative thermograms (DTG) showing the rate of weight loss (derivative of residual weight with temperature) as a function of temperature during the heating of the sample at a constant heating rate (10 °C/min). It could be seen that the homopolymer P(3HB) was degraded in a single step process, while the P(3HB-*co*-4HB) copolymers were decomposed in two steps. However, these two steps did not represent separate decomposition of 3HB and 4HB units as it was sometimes incorrectly referred [30], because the two-step thermal degradation was reported also for the P(4HB) homopolymer [28]. In fact, the difference in thermal degradation dynamics illustrated different mechanisms of the decomposition processes. While P(3HB) was degraded primarily by *cis*-elimination reaction releasing crotonic acid as a volatile product [31], P(4HB) at first underwent an unzipping reaction from the ω-hydroxyl end, followed at higher temperatures by cyclic rupture via intramolecular transesterification [32]. In P(3HB-*co*-4HB) copolymers, all these mechanisms took place at the rates affected by the particular chemical composition. Nevertheless, it could be generalized from the DTG curves in Figure 5 that the incorporation of 4HB monomers in the polymer resulted in a gradual increase in thermal stability. This fact was even more obvious from the comparison of the temperatures of half-decomposition (T_50%_) presented in Figure 6. Moreover, it could be seen from the results shown in Figure 5 and Figure 6 that the P(3HB-*co*-4HB-*co*-3HV) terpolymer PHA9 was decomposed in three stages and its T_50%_ was almost as high as that for a copolymer with 90% 4HB.

Another important advantage of the incorporation of 4HB monomers into the P(3HB) matrix arose from discussion of the results of DSC and TGA analyses. It could be seen that 4HB both lowered the melting point and increased the temperature of decomposition. Therefore, it widened the polymer processing temperature window, where the polymer in melt could be processed without serious thermal degradation. This is a key improvement from the perspective of polymer processing technology.

**Figure 7:**
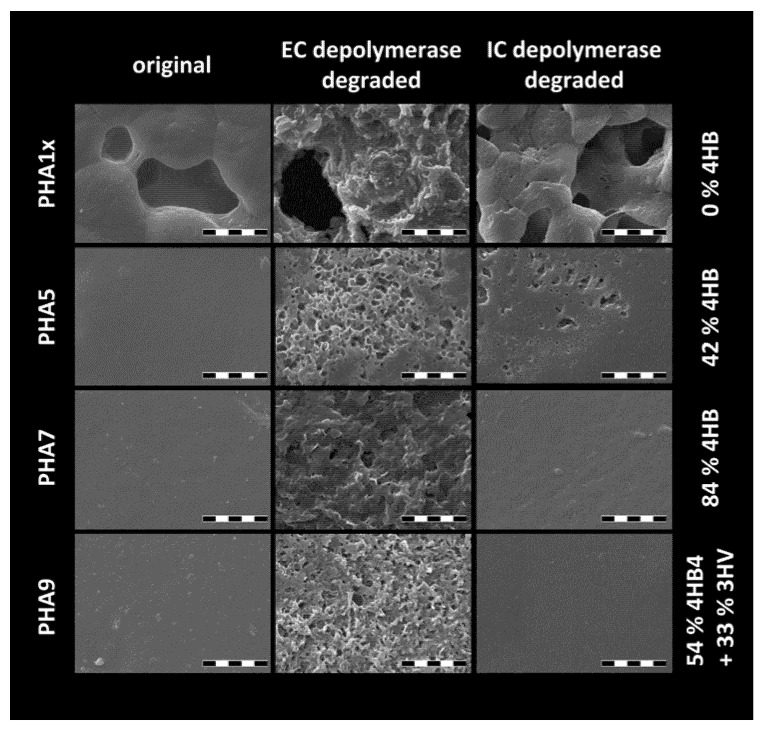
SEM micrographs of PHA copolymer films before and after the enzymatic degradation assay using extracellular (EC) PHA depolymerase and intracellular (IC) PHA depolymerase, respectively (magnification 10,000×, scalebar: 10 µm).

Finally, biodegradability as another essential application-relevant property of the PHA based material was checked by a preliminary enzymatic degradation assay. Both extracellular and intracellular PHA depolymerases were used for this purpose in parallel because it has been well-described that the two types of enzymes differ in their specificity to ordered and disordered PHA structures, respectively [33]. Because the film thickness was not controlled during solvent-casting, the degradation process was not followed by measurement of the weight losses over time. Instead, biodegradation was screened by directly visualizing changes in surface topography with scanning electron microscopy (SEM), which provided preliminary information about assessment of PHA films to biodegradation by a particular depolymerase. As can be seen in Figure 7 (and in Figure S3 on a smaller scale), surface topography differed significantly even for the as-prepared polymer films. High crystallinity of homopolymer P(3HB) (PHA1x) resulted in porosity caused by the sample shrinkage during film preparation. Consequently, the large specific surface of the porous P(3HB) film resulted in increased contact area with the enzymatic solution and more significant degradation even for intracellular depolymerase, which specifically decomposes amorphous PHA domains. The porous character of the film was to some extent also preserved for other polymers with a dominant content of 3HB (PHA2x, PHA3x), where the pore surface obviously constituted the center of the enzymatic attack. For the copolymers with a lower 3HB content, original films had a smooth surface with no apparent effect of specific polymer composition.

Among the individual PHA copolymers, conclusive signs of changes in surface structure as a direct indicator of degradation by extracellular PHA depolymerase were detected for all samples, however, from the qualitative comparison of the extent of surface degradation it seemed that the polymers with the highest content of 4HB were the most prone to decomposition. This represents an unexpected observation, which is in contradiction with the study of Saito et al. [34], who reported a higher rate of degradation by depolymerase from *Alcaligenes faecalis* at 37 °C for P(3HB-*co*-4HB) copolymers with low and intermediate content of 4HB compared to P(3HB), but higher resistance to the enzymatic degradation of P(4HB) homopolymer and copolymers with 4HB content higher than about 80%. Nevertheless, for a conclusive confirmation of this phenomenon, a more systematic follow-up investigation of the enzymatic degradation of materials tested in this study is needed. Concerning degradation of the copolymer films by intracellular depolymerase, films prepared from polymers with a higher content of 4HB (PHA6, PHA7 and PHA8) and from the terpolymer (PHA9) showed obviously higher stability than porous films with the highest content of 3HB (PHA1x, PHA2x, PHA3x) and also compared to the films with intermediate content of 4HB (PHA4, PHA5), where the degradation was evidently supported by lower crystallinity of the material. 

As already noted, the degradation assay involved in this study was intended as a preliminary screening test, serving for a qualitative comparison of degradation behavior among the tested PHA materials. In a follow-up study, continuous monitoring of quantitative parameters accompanying the degradation process (weight loss, changes in film thickness, decrease in average molecular weight) will not only describe degradation kinetics on higher resolution, but it will also enable correlation of the material’s stability with other quantitative chemical (monomer content, Mw) and physical parameters (degree of crystallinity) and thus help to understand the exact mechanism of the decomposition.

## 4. Conclusions

In this study, PHA polymers produced by the newly isolated, moderately thermophilic Gram-positive bacterium, *Aneurinibacillus* sp. H1, were subjected to an in-depth material analysis. With respect to the outstanding capability of the strain to change the monomer composition of accumulated PHA according to the cultivation conditions, we focused this initial study primarily on the way the relative content of 3HB and 4HB monomers affected the degree of structural order and basic physicochemical parameters of solvent-casted films prepared from the isolated PHA polymers. In order to cover the whole range of monomer compositions in this comparative study, reference P(3HB) homopolymer and P(3HB-*co*-4HB) copolymers with minor 4HB content were prepared by routinely used mesophilic PHA producer *C. necator* H16.

The results of molecular spectroscopy, XRD and DCS assays demonstrated clearly that in the 3HB prevailing polymers, the 4HB monomer acted as a structure-breaking component of P(3HB) crystalline lattice and, similarly, when the 4HB monomer predominated the polymer composition, residual 3HB units reduced the overall P(4HB) crystal lattice content. Consequently, polymers with a comparable content of 3HB and 4HB in the polymer chain showed the lowest crystallinity and the amorphous character of the material predominated. Presence of 3HV in terpolymer virtually amplified the effects of 4HB (the material behaved like it had a higher 4HB content). In this way, it was confirmed experimentally that adjusting of the monomer composition of the polymer enabled optimization of its crystallinity both qualitatively (the type of the crystalline lattice) and quantitatively (the overall degree of crystallinity).

Moreover, the results of the combination of thermoanalytical methods used (DSC and TGA) showed that while the incorporation of a 4HB monomer in the polymer composition lowered the melting temperature significantly, it also resulted in a gradual increase in stability against thermal decomposition. P(3HB-*co*-4HB) copolymers were thus less prone to thermal degradation during melt processing, which represented a key improvement from the perspective of polymer processing technology. Furthermore, qualitative results provided by the preliminary enzyme-degradation assay indicated that manipulating the monomer composition of the copolymers affected the rate of its biodegradation.

To sum up, our study confirms that the isolated bacterium *Aneurinibacillus* sp. H1 represents a highly promising candidate for the biotechnological production of PHA not only from the economical point of view (as discussed in detail in the preceding paper in this series), but also with respect to its capability to produce polymers with material properties that can be tuned together with their chemical composition by the corresponding adjustment in the cultivation process. In order to evaluate the ability of PHA polymers produced by *Aneurinibacillus* sp. H1 to meet specific requirements of particular application areas, further material analysis focused on mechanical performance, transport properties (gas and solute permeabilities, etc.) and stability in various environments must be supplemented in a follow-up study.

## Figures and Tables

**Table 1:** Solvent-casted films from polyhydroxyalkanoates (PHA) copolymers analyzed in the study.

Sample Identification	Bacterial Production Strain	Monomer Composition			Mw [kDa]	PDI
		3HB	4HB	3HV		
		[mol. %]	[mol. %]	[mol. %]		
PHA1x	*C. necator* H16	100%	–	–	324 ± 4	1.18
PHA2x	*C. necator* H16	98.50%	1.50%	–	231 ± 2	1.18
PHA3x	*C. necator* H16	93.50%	6.50%	–	225 ± 4	1.14
PHA4	*Aneurinibacillus* sp. H1	64%	36%	–	66 ± 1	1.59
PHA5	*Aneurinibacillus* sp. H1	58%	42%	–	76 ± 1	1.78
PHA6	*Aneurinibacillus* sp. H1	26%	74%	–	86 ± 1	1.32
PHA7	*Aneurinibacillus* sp. H1	16%	84%	–	120 ± 1	1.27
PHA8	*Aneurinibacillus* sp. H1	10%	90%	–	128 ± 4	1.29
PHA9	*Aneurinibacillus* sp. H1	13%	54%	33%	109 ± 4	1.28

## Supplementary Materials

The following are available online at https://www.mdpi.com/2073-4360/12/6/1298/s1, Figure S1: Visual appearance of the PHA films investigated in this study. Figure S2: Complete FTIR spectra of the solution-casted PHA films prepared from polymers with different monomer composition (each spectrum represents an arithmetic average of 10 individual spectra collected at different locations on a surface of the film). Figure S3: SEM micrographs of PHA copolymer films before and after the enzymatic degradation assay (magnification 1000×, scalebar: 100 µm).
