# Supplementary material for: Introducing the Newly Isolated Bacterium Aneurinibacillus sp. H1 as an Auspicious Thermophilic Producer of Various Polyhydroxyalkanoates (PHA) Copolymers–2. Material Study on the Produced Copolymers
Source: Polymers (Basel). 2020 Jun 5;12(6):1298. doi: 10.3390/polym12061298 (PMC7362046; doi:10.3390/polym12061298)
Supplement: Supplementary file 1 [file polymers-12-01298-s001.pdf]

Supplementary material to:

Introducing the newly isolated bacterium *Aneurinibacillus* sp. H1 as an auspicious thermophilic producer of various PHA copolymers – 2. Material study on the produced copolymers

Petr Sedlacek[1], Iva Pernicova[1], Ivana Novackova[1], Xenie Kourilova[1], Michal Kalina[1], Adriana Kovalcik[1], Martin Koller[2,3], Jana Nebesarova[4,5], Vladislav Krzyzanek[6], Kamila Hrubanova[6], Jiri Masilko[1], Eva Slaninova[1], Monika Trudicova[1], Stanislav Obruca[1]*

1 Faculty of Chemistry, Brno University of Technology, Purkynova 118, 612 00 Brno, Czech Republic.
2 Institute of Chemistry, NAWI Graz, University of Graz, Heinrichstrasse 28/VI, 8010 Graz, Austria.
3 ARENA Arbeitsgemeinschaft für Ressourcenschonende & Nachhaltige Technologien, Inffeldgasse 21b, 8010 Graz, Austria.
4 Biology Centre, The Czech Academy of Sciences, v.v.i., Branisovska 31, 370 05 Ceske Budejovice, Czech Republic.
5 Faculty of Science, University of South Bohemia, Branisovska 31, 370 05 Ceske Budejovice, Czech Republic.
6 Institute of Scientific Instruments of the Czech Academy of Sciences, v.v.i., Kralovopolska 147, 612 64 Brno Czech Republic.

* Correspondence: obruca@fch.vut.cz; Tel.: +420 541 149 354

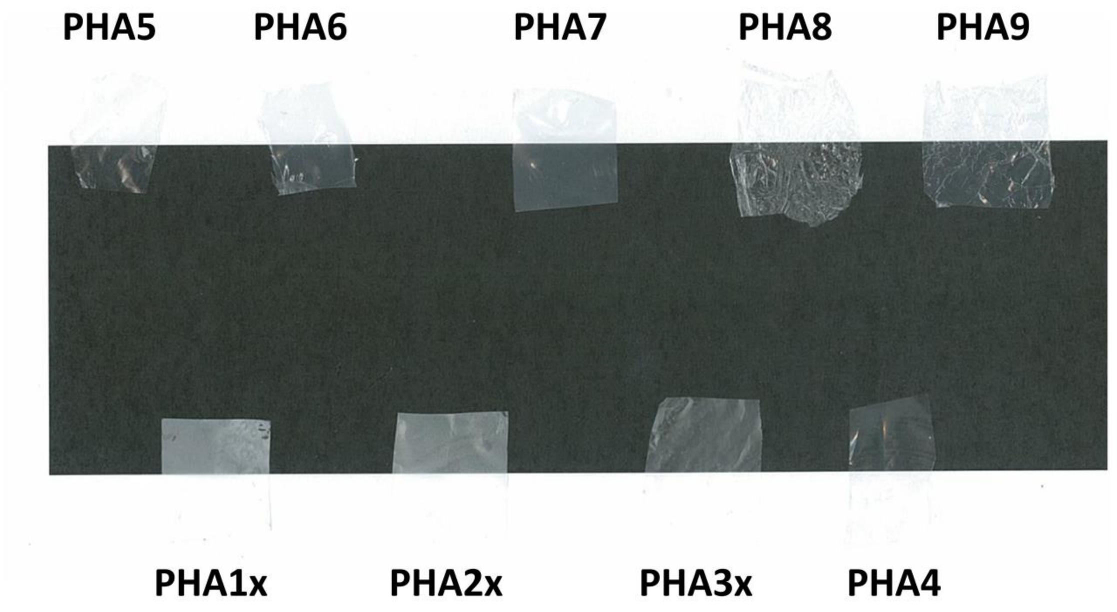

Figure S1 Visual appearance of the solvent-casted PHA films involved in the study. Legend to the sample identification is provided in Tab. 1.

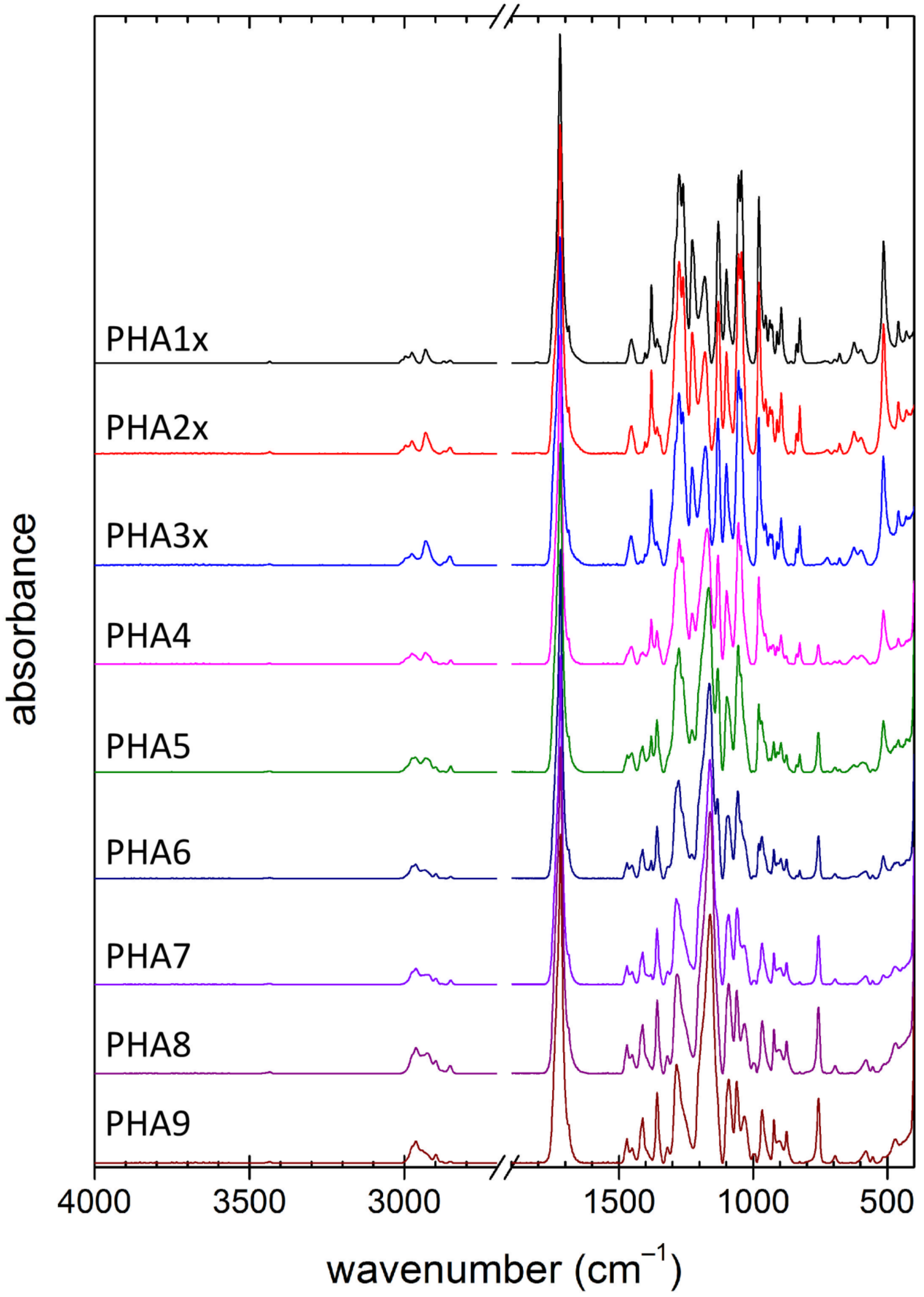

Figure S2 Complete FTIR spectra of the solution-casted PHA films prepared from polymers with different monomer composition (each spectrum represents an arithmetic average of 10 individual spectra collected at different locations on a surface of the film).

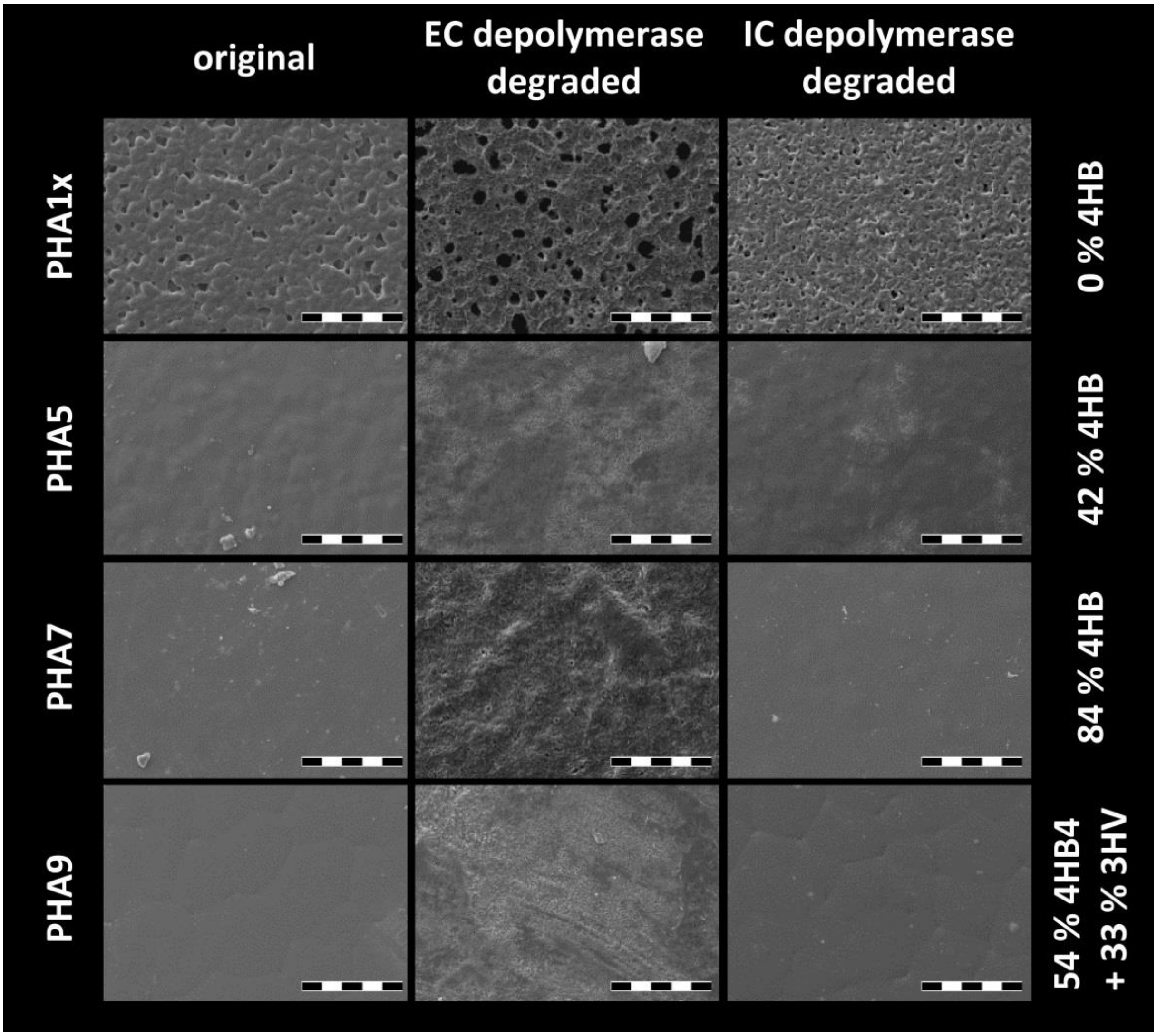

Figure S3 SEM micrographs of PHA copolymer films before and after the enzymatic degradation assay (magnification 1,000×, scalebar: 100 μm).
